# Transcriptional regulation of *GmNAC3*-mediated drought stress tolerance in soybean

**DOI:** 10.1080/21645698.2025.2516295

**Published:** 2025-06-24

**Authors:** Nooral Amin, Liu Lu, Faizur Rehman, Muhammad Imran, Gai Yuhong, Piwu Wang, Wei Jian

**Affiliations:** a Plant Biotechnology Centre, College of Agronomy, Jilin Agricultural University, Changchun, China; b School of Breeding and Multiplication (Sanya Institute of Breeding and Multiplication), School of Tropical Agriculture and Forestry, Hainan University, Sanya, China

**Keywords:** Agrobacterium-rhizogenes, drought tolerance, *GmNAC3*, hairy roots, overexpression, soybean

## Abstract

Drought stress is a major limiting factor that adversely affects both the yield and quality of soybean crops. Transcription factors (TFs) play a pivotal role in regulating gene expression, facilitating plant adaptation and response to various abiotic stresses. Among the 179 *NAC* TFs encoded in the soybean genome, several are differentially expressed under stress conditions; however, the functional role of *GmNAC3* in drought tolerance remains largely unknown. In this study, we cloned the 840 bp coding sequence of *GmNAC3* and developed transgenic soybean hairy roots via *Agrobacterium*-mediated transformation to explore its role in drought response. The physiological and molecular responses of *GmNAC3* overexpression (OE) chimeric soybean plants were assessed under polyethylene glycol (PEG)-simulated drought stress using Hoagland nutrient solution. Compared to empty vector (EV) controls, OE plants exhibited enhanced drought tolerance, including improved phenotypic traits, better root development, and stress resilience. Notably, OE plants showed a 23.9% reduction in hydrogen peroxide accumulation and a 31.25% decrease in superoxide anion levels. Biomass analysis on MS medium revealed significantly higher fresh and dry weights of OE hairy roots across different mannitol concentrations compared to EV roots. Furthermore, *GmNAC3* overexpression led to the upregulation of key downstream genes involved in stress response, particularly *GmLAC5* and *GmLAC7*. These findings suggest that *GmNAC3* enhances drought tolerance in soybean by regulating both physiological and molecular pathways. Overall, GmNAC3 represents a promising target for genetic engineering aimed at improving drought resistance in soybean and potentially other crops.

## Introduction

Soybean (*Glycine max* L.) is an important crop, widely used for both human consumption and livestock feed, while also playing a significant role in various industrial applications due to its high protein and oil content.^1^ The global soybean production was reported to have reached 338.6 million tons.^2^ However, the productivity of soybean is severely affected by both biotic and abiotic stresses, with drought being the most detrimental. Water scarcity during the reproductive stage significantly disrupts plant development by inducing flower abscission, embryo abortion, and reduced pod formation, which collectively contributes to decreased seed number and size.^3^ Understanding the roles of genes that regulate developmental processes and stress responses in plants is important for adjusting crop yields.^4^ Transcription factors play a pivotal role in how plants adapt to environmental stresses, and families such as WRKY, DREB, MYB, and NACs are central to mediating these responses. In particular, NAC transcription factors (TFs) play a pivotal role in orchestrating plant stress responses and have been widely recognized for their contribution to improving crop resilience under adverse environmental conditions.^5,6^ These TFs regulate gene expression during stressful conditions, which is essential for stress adaptation. The NAC TF family is implicated in diverse developmental processes, including the regulation of shoot apical meristem development,^7,8^ leaf senescence, cell wall formation, and both abiotic and biotic stress factors.^9–11^ NAC family members possess a conserved domain structure, including the NAM, ATAF1/2, and CUC2 domains.^12^ Comprehensive genome sequencing has revealed 151 NAC TFs in rice and 126 in *Arabidopsis* .^13^ In *Arabidopsis thaliana*, multiple NAC genes have been shown to play critical roles in mediating plant responses to various environmental stress conditions. For example, *ANAC032*, an abscisic acid-responsive NAC TF, is induced by oxidative stress and abiotic stimuli such as high light intensity, osmotic stress, and salinity. This activation reduces anthocyanin production and promotes leaf senescence.^14,15^ The overexpression of NAC TFs, such as *NAC019* and *NAC055* have been shown to enhance drought tolerance in *Arabidopsis*.^16^ Similarly, overexpression of the NAC gene *ATAF1*, which is induced by abiotic stress, leads to dwarfism and inhibits primary root growth.^17^ *ATAF1* overexpression in rice improves salt tolerance and increases sensitivity to abscisic acid (ABA).^18^ Extensive research on *NAC* genes has revealed that *GmNAC3*0 and *GmNAC81* play critical roles in regulating stress-induced programmed cell death in soybean.^19,20^ Additionally, *GmNAC004, GmNAC11*, and *GmNAC20* have been shown to be involved in the plant’s response to external stresses and the regulation of lateral root formation and development.^20,21^

Further research has explored expression profiles of *GmNAC019* and *GmNAC109* in different soybean tissues from both drought-tolerant and sensitive cultivars under drought stress has demonstrated these TFs as a promising target for enhancing drought resilience.^22^ Despite the valuable insights gained into their functions, only a limited number of *GmNAC* family members in soybean have been thoroughly studied, and additional research is needed to clarify their specific and overlapping roles in stress tolerance. This study focuses on *GmNAC3* (Glyma.01G051300), a gene previously identified as a candidate for drought resistance in *Arabidopsis*.^5^ To investigate the functional role of *GmNAC3 in* soybean, we constructed a binary expression vector and introduced it into soybean hairy roots. Under drought conditions, the resulting chimeric plants demonstrated improved stress tolerance and showed superior performance compared to non-transgenic controls. Comprehensive phenotypic, physiological, and molecular analyses provided valuable insights into the functional role of *GmNAC3* in enhancing drought tolerance in soybean. qRT-PCR analysis of downstream target genes in the chimeric soybean plants revealed significant changes in expression levels associated with *GmNAC3* overexpression. Our findings indicate that *GmNAC3* functions as a transcription factor that contributes to the drought stress response by regulating the expression of downstream drought-responsive genes, underscoring its potential as a candidate for developing drought-tolerant soybean cultivars.

## Materials and Methods

### Gene Cloning and Construction of Plant Overexpression Vector

The complete coding sequence (CDS) of *GmNAC3* (840 bp) was amplified by PCR using cDNA as the template. Primers were designed to incorporate with BamHI and HindIII restriction sites to facilitate directional cloning. For PCR amplification, the forward primer was NAC-F: 5’ ACACGGGGGACTCTAGAATGGAGAATAGAACAAGCTCTG 3,” and the reverse primer was NAC-R: 5” GGAGGACCTCTAGATGCATAAGGGATTTCAACCA GC 3’ using Pfu DNA polymerase (Takara, China). The PCR amplification process followed these thermal cycling conditions: initial denaturation at 95°C for 1 minute, followed by 35 cycles of denaturation at 94°C for 30 seconds, annealing at 56°C for 30 seconds, and extension at 72°C for 1 minute and 30 seconds, with a final extension at 72°C for 10 minutes. The amplified gene fragment was then ligated into the pEASY-T1 cloning vector and introduced into competent cells via the heat-shock method.^23^ Positive transformants were first screened out and then verified by sequencing. After confirmation, the target gene was excised from the cloning vector using EcoRI and BamHI restriction enzymes and inserted into the pCAMBIA3301 overexpression vector, which contains a glyphosate resistance gene (Bar). The recombinant plasmid was then transformed into *E. coli* DH5α competent cells following the above-described method, and positive transformants were verified using colony PCR, followed by further sequencing for confirmation. Finally, the Expression construct pCAMBIA-*NAC3* was introduced into the *Agrobacterium rhizogenes* strain K599, and the transformed strain was stored in glycerol at −80°C for further use.

### Soybean Hairy Root Transformation via Agrobacterium Rhizogenes

The soybean hairy root system is a widely used technique for functional gene analysis.^24^ To develop the hairy roots, high-quality seeds of the William 82 soybean cultivar were selected and sterilized using chlorine gas for 16 hours. The sterilized seeds were then planted in pots containing sterilized vermiculite and maintained in a growth chamber at 28°C with a 16-hour photoperiod and watered regularly to ensure optimal growth conditions. At the same time, the Agrobacterium rhizogenes strain K599 was streaked onto YEP agar plates containing kanamycin and incubated at 28°C for 52 hours. The inoculum was then cultured in a shaker at 250 rpm for 14 hours at 28°C. A 150 μL sample of the culture was transferred onto YEP plates containing kanamycin and incubated at 28°C for another 46 hours.

### Regeneration and Verification of Transgenic Hairy Roots

Once the soybean seedlings were began to sprout, five-day-old seedlings were used for infection with the Agrobacterium rhizogenes K599 strain carrying pCAMBIA-*NAC3* recombinant construct. The Agrobacterium culture was collected from the petri-plate subculture and introduced into the hypocotyls of young soybean plants at the base of the cotyledons, following the procedure of Kereszt el.^25^ The K599 strain containing the empty vector was used to regenerate hairy roots for the control group (Empty Vector, EV). After transformation, the seedlings were placed in polytene trays to maintain optimal humidity. Approximately 15 days after transformation, the hairy roots began to emerge at the infection sites. The seedlings with established hairy roots were allowed to grow well for an additional two weeks. Following the complete development of hairy roots, the presence of the *Bar* gene in transgenic roots (both EV and OE lines) was initially confirmed using a Bar rapid strip test. Plants that tested positive were subsequently validated by PCR analysis. Additionally, the EV and OE roots were scanned using a root scanner, and the data were analyzed using appropriate software tools.

### Drought Evaluation of Transgenic Hairy Roots in Hoagland-PEG

Once the soybean hairy roots reached a length of 8–10 cm, the plants were carefully uprooted, and the primary roots were removed. The resulting chimeric plants, possessing transgenic roots and non-transgenic shoots, were then transplanted into new pots containing sterilized vermiculite. These plants were watered regularly to maintain high humidity levels. After the emergence of the second trifoliate leaf pair, the shoots of each plant were pruned. The newly formed leaves of both the chimeric EV and OE transgenic plants were then compared. To assess the drought tolerance of the transgenic hairy roots, both EV and OE plants, with the same hairy root’s length were transferred to Hoagland solution. Following a 4-day acclimatization period, the plants were subjected to 6% PEG6000 treatment to simulate drought stress.

### Determination of O₂^−^ Content in Chimeric Soybean Leaves

Both EV and OE chimeric plants were exposed to drought stress. The presence of superoxide anion (O₂^−^) and hydrogen peroxide (H₂O₂) in the leaves under normal and drought conditions was assessed through histochemical staining using nitro blue tetrazolium (NBT) and 3,3’-diaminobenzidine (DAB), respectively.^26,27^ To detect O₂^−^ levels, leaves from both EV and OE chimeric plants were immersed in a 1 mg/mL NBT solution (pH 7.8) at room temperature in the dark for 3–4 hours. For H₂O₂ detection, the leaves were submerged in a 1 mg/mL DAB solution (pH 3.8) for 6–8 hours. After staining, the leaves were washed with sterile water, then decolorized by immersing them in 95% ethanol and heated in a water bath at 75°C for 20–25 minutes. To quantify O₂^−^ and H₂O₂ levels, the stained leaves were homogenized in potassium phosphate buffer, incubated, and centrifuged at 12,000 rpm for 10 minutes. The obtained supernatant was analyzed using a spectrophotometer, with absorbance measured at 530 nm for O₂^−^ and 485 nm for H₂O₂, and concentrations were determined based on standard curves.^28^ These absorbance values were reflected as the levels of superoxide radicals and hydrogen peroxide in the leaf tissues.

### Transgenic Hairy Root Growth Assessment on MS Medium Supplemented with Mannitol

Approximately 0.1 g of EV and OE hairy roots were carefully weighed under sterile conditions and cultured on germination medium supplemented with various mannitol concentrations (0 mm, 50 mm, 100 mm, 150 mm, and 200 mm). After a 12-day incubation period, the fresh and dry weights of the chimeric hairy roots were measured. The dry weight was obtained by drying the root samples overnight at 58°C.

### Examination of Physiological Parameters

Proline and malondialdehyde (MDA) levels, along with catalase (CAT) activity and relative electrolyte leakage (REL), were assessed in both EV and OE roots following the manufacturer’s protocol. The proline content was determined following the method of Bates et al.^29^ Malondialdehyde (MDA) levels, an indicator of lipid peroxidation, were quantified using the thiobarbituric acid (TBA) assay as described by Hodges et al.^30^ CAT activity was quantified using the method described by.^31^ In the reaction mixture, 50 μL of enzyme solution was added to 3 mL of a buffer containing 2.4 mL of 100 mm potassium phosphate (pH 7.0) and 0.6 mL of 100 mm hydrogen peroxide (H₂O₂). Colorimetric measurements were made at 240 nm, and readings were taken at 30-second intervals for a total of five measurements. The CAT enzyme activity was quantified based on the reduction in absorbance per minute. Relative electrolyte leakage (REL) was measured using the method outlined by Ahmad et al.^32^ Initially, the electrical conductivity (ECa) of a 2 g sample was measured after it was rinsed and submerged in 10 mL of sterile water. The samples were then incubated at 55°C for 25 minutes, followed by the measurement of ECb. Finally, the samples were boiled for 12 minutes, and the ECc was measured. The electrolyte leakage percentage was calculated using the formula: % = (ECb – ECa)/ECc × 100.

### *Expression Analysis of the* GmNAC3 *Regulatory Network in Soybean*

The STRING database was utilized to predict potential interactors of *GmNAC3* in soybean. qRT-PCR primers targeting six selected genes were designed using Primer-BLAST, primers are listed in Table S1. To assess the impact of *GmNAC3* overexpression on its interacting proteins, RNA was extracted from both EV and OE samples using RNAiso Plus (Takara). High-quality total RNA was transcribed into cDNA using the Takara reverse transcription kit. qRT-PCR was conducted in triplicate, Actin 11 used as an internal control. the relative expression levels of target genes were calculated using the 2-^ΔΔ^Ct method.

### *Subcellular Localization of* GmNAC3 *via Transient Expression in Onion Epidermal Cells*

To examine the subcellular localization of GmNAC3, transient transformation was performed in onion epidermal cells.^33^ The complete coding sequence of GmNAC3 was inserted into the pVBG2307-GFP vector to establish the pVBG2307-GmNAC3-GFP recombinant construct. This construction, driven by the constitutive 35S promoter from cauliflower mosaic virus (CaMV), was introduced into competent *Agrobacterium tumefaciens* strain GV3101. The transformed strain was collected by centrifugation and resuspended in 10 mm MES (pH 5.5), 10 mm MgCl₂, and 200 μM acetosyringone (AS) solution. The final bacterial suspension had an OD600 of 0.8–1.0. Onion epidermal cells were infiltrated with the Agrobacterium suspension for 30 minutes and incubated in the dark for 30 hours. The same procedure was applied to control cells infiltrated with the empty vector. After incubation, the cells were examined using an OLYMPUS B×63 automated fluorescence microscope (OLYMPUS, Japan) to assess the localization of the GFP-tagged protein.^34^

### Statistical Analysis

Statistical analyses were performed using SPSS software (version 25.0). Each experiment was replicated three times for the analysis of variance (ANOVA). Statistical significance was determined at *p* < .05, indicated by different small letters on the bars. Tukey’s test and the Student’s t-test were used to compare the means. Additionally, data visualization was conducted using GraphPad Prism version 10.2.1.

## Results

### *Overexpression of* GmNAC3 *in Soybean Hairy Roots*

**Figure 1.**
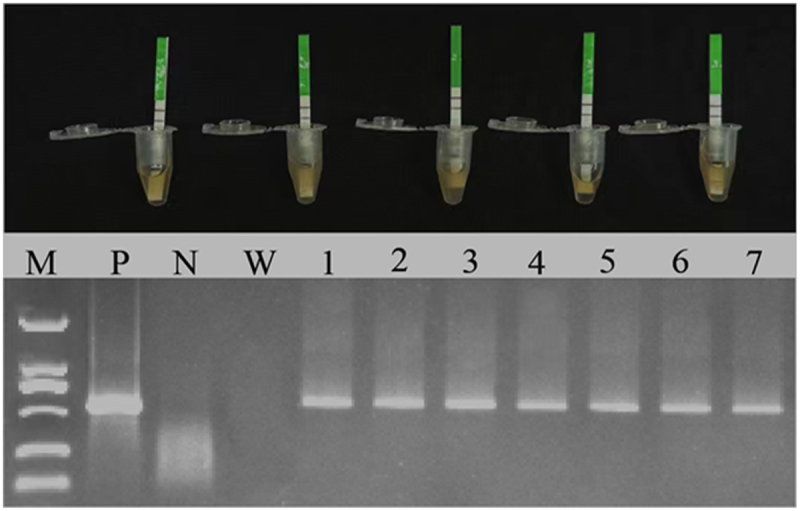
Detection of the K599 transformants carrying the OE construct. Confirmation of different transgenic roots with the Bar rapid strip. Further validation was performed with the Bar gene(552-bp) via PCR, where *M* = 2000bp, P: positive control, N: negative control, W: wild type, lanes: 1 to 7 represent different transgenic roots.

To validate the role of *GmNAC3* in drought stress response, a soybean hairy root transformation system was used to achieve overexpression of *GmNAC*. After 2 weeks of transformation, soybean seedlings with K599 derived hairy roots (EV and OE) began to regenerate from infection sites (Figure S2). Prior to further experimentation, the presence of the transgene was confirmed using a Bar rapid strip test, and DNA was extracted from a small portion of the hairy roots to verify the transgenic roots through PCR amplification using Bar gene specific primers (Figure 1). Once confirmed, the transgenic roots, along with non-transgenic shoots, were transplanted into fresh pots containing autoclaved vermiculite.

### Drought Tolerance of Chimeric Soybean with OE Hairy Roots

**Figure 2.**
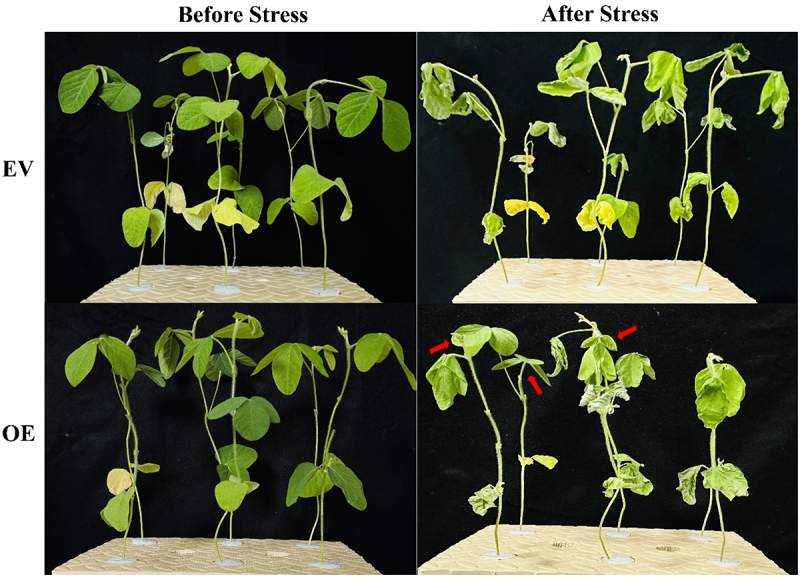
Drought analysis of chimeric soybean plants. Soybean plants with EV (Empty vector) and *GmNAC3*-OE roots were subjected to 6% PEG6000 stress in a Hoagland solution. Images were taken both before and after the stress treatment to evaluate their drought tolerance. The fresh leaves of OE plants were less affected by PEG stress compared to EV plants, as indicated by red arrows.

When the transgenic roots reached approximately 8–10 cm, both EV and OE soybean plants were transferred from the vermiculite to a hydroponic Hoagland solution (Figure 2). After a 4-day acclimatization period, both EV and OE plants were subjected to drought conditions using 6% PEG6000. Upon exposure to drought, both groups exhibited leaf curling and drooping shoot tips. However, OE plants displayed milder symptoms with reduced leaf curling and wilting of the aerial leaves compared to EV plants (Figure 2). These observations indicate that overexpression of *GmNAC3* in the hairy roots enhances drought tolerance in the chimeric soybean plants, potentially improving their resilience under stress.

### Histochemical Analysis and Superoxide (O₂^−^) Levels in Soybean Leaves

**Figure 3.**
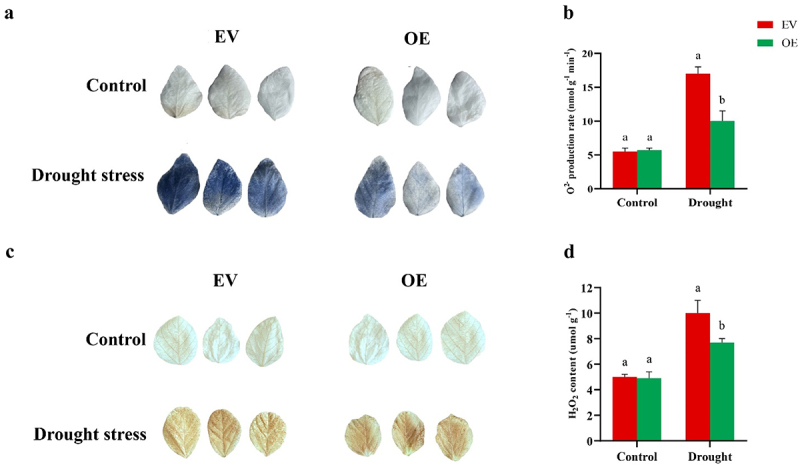
Histochemical assay of the leaves of chimeric soybean. The leaves of the soybean plants (EV and OE) were stained before and after drought stress. (a, b) NBT staining assay and superoxide anions quantification leading to the formation of an insoluble formazan complex. (c, d) represents DAB staining assay and quantification of H2O2.

Plants produce higher levels of reactive oxygen species (ROS) under stressful conditions and activate both enzymatic and non-enzymatic mechanisms to mitigate excess ROS accumulation. To explore the role of *GmNAC3* under drought stress, the leaves of 6-week-old chimeric plants (EV and OE) were stained with NBT and DAB before and after 24 hours of PEG-induced stress to visualize in situ accumulation of H₂O₂ and O₂^−^. The OE chimeric plants exhibited greater tolerance to drought stress than EV plants. Under normal conditions, no significant differences were observed between EV and OE leaves; However, following drought stress treatment, a substantial increase in ROS levels was detected in both OE and EV leaves. Notably, the EV leaves accumulated significantly higher levels of H₂O₂ and O₂^−^, as indicated by the intense brown (Figure 3a) and blue staining (Figure 3c). Similarly, the OE lines exhibited lower levels of H₂O₂ and O₂^−^ compared to the EV plants (Figure 3b,d). These results demonstrate that overexpression of *GmNAC3* enhances drought tolerance by mitigating ROS accumulation in soybean chimeric plants.

### Physiological Assessments of OE Hairy Roots

**Figure 4.**
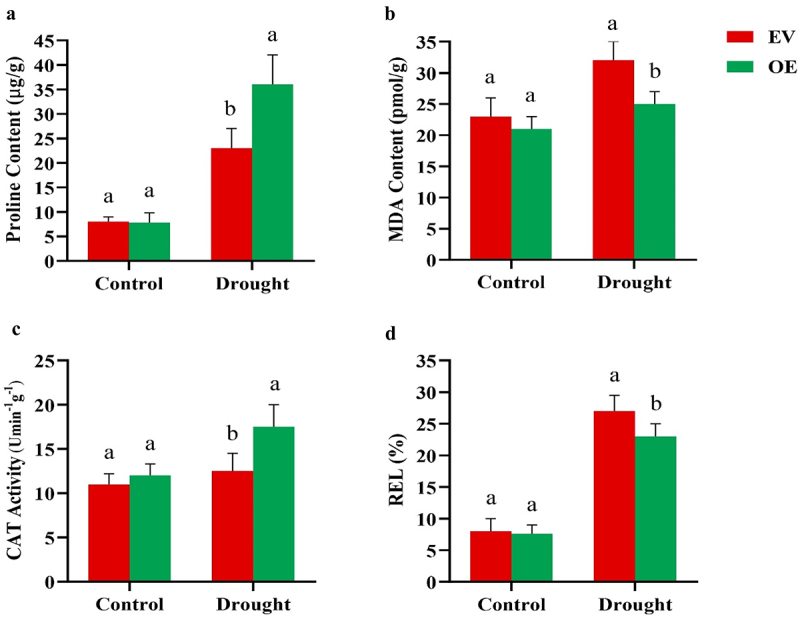
Physiological profiles of chimeric plants. Soybean chimeric plants exposed to drought conditions were evaluated for (a) proline levels, (b) MDA content, (c) CAT activity, and (d) REL activity. Data are presented as means ± SD (*n* = 3), with different letters on the bars indicating significant differences (*p* < .05).

To evaluate the physiological effects of *GmNAC3* overexpression in chimeric plants, proline levels, MDA (malondialdehyde) content, CAT (catalase) activity, and electrolyte leakage (REL) were measured in both EV and OE hairy roots. Under control conditions, proline levels were similar in both EV and OE roots (Figure 4a). However, during PEG-induced stress, proline levels were notably higher in OE roots. Similarly, the MDA content, which is an indicator of lipid peroxidation and membrane damage, was lower in OE roots compared to EV roots, especially under drought conditions (Figure 4b). Moreover, CAT activity was markedly higher in OE roots than in EV roots under stress conditions (Figure 4c). Electrolyte leakage, reflecting cell membrane integrity, was significantly reduced in OE roots compared to EV roots under drought stress (Figure 4d). These results suggest that overexpression of *GmNAC3* enhances the drought tolerance of OE roots by optimizing physiological processes.

### Growth Comparison of EV and OE Hairy Roots on MS-Mannitol Medium

**Figure 5.**
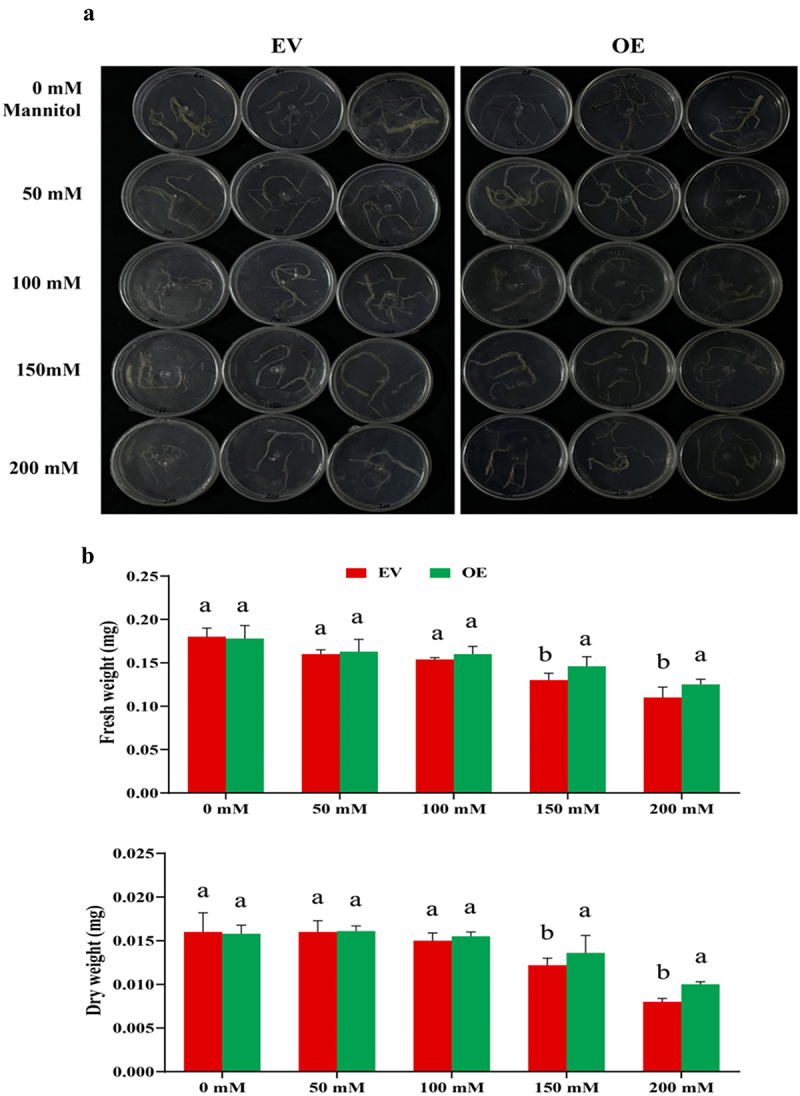
Analysis of fresh and dry weight in transgenic hairy roots. (a) 0.1 g of EV and OE roots were cultured on MS media containing varying concentrations of mannitol (50, 100, 150, and 200 mm) along with a control (0 mm) for 12 days to assess their growth performance under drought-simulating conditions. (b) Fresh weight and dry weight.

To assess the growth efficiency under drought conditions, EV and OE hairy roots were cultured on germination medium supplemented with different concentrations of mannitol (0 mm, 50 mm, 100 mm, 150 mm, and 200 mm). After 12 days of incubation, the fresh and dry weights of the roots were measured. While the control roots (0 mm mannitol) showed similar fresh and dry weights between EV and OE hairy roots, conversely, the OE roots demonstrated significantly better growth under mannitol stress, particularly at 150 mm and 200 mm concentrations than EV roots (Figure 5a). All the samples were incubated in the dark at 28°C in a growth room for 12 days. After this period, the fresh and dry weights were measured. The control samples of both EV and OE hairy roots exhibited similar fresh and dry weights. However, under stress conditions with mannitol, the OE hairy roots outperformed to EV roots, showing significantly higher weights at 150- and 200-mM mannitol concentrations (Figure 5b). This indicates that the overexpression of *GmNAC3* improves drought tolerance in the roots under osmotic stress.

### Phenotypic Analysis of OE Hairy Roots

**Figure 6.**
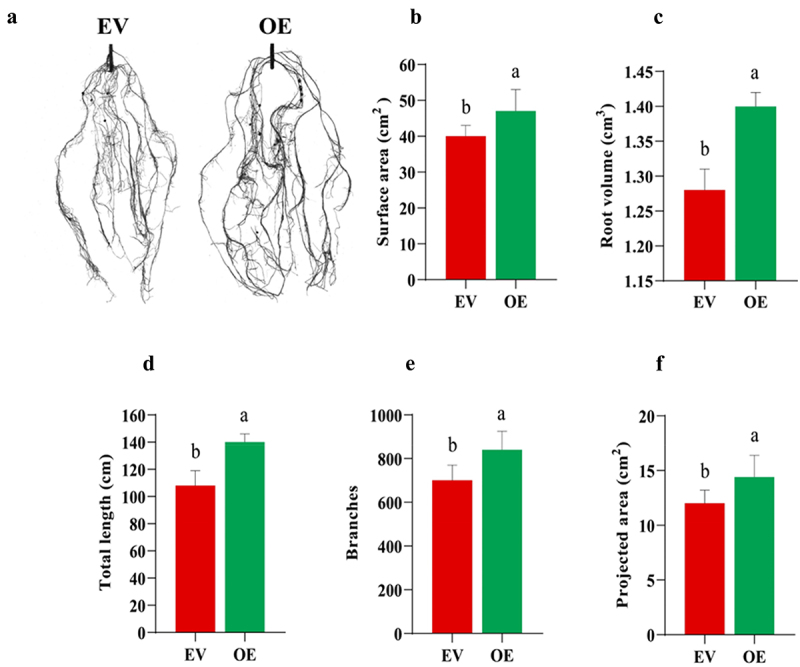
Phenotypic assessment of EV and OE soybean roots. (a) Hairy roots of both types were examined by operating a root scanner. Measurements contained (b) surface area, (c) root volume, (d) total length, (e) number of branches, and (f) projected area of the EV and OE roots.

Root scanning analysis showed that OE roots exhibited superior growth metrics compared to EV roots (Figure 6a). Specifically, OE roots had a larger total surface area (Figure 6b), greater root length (Figure 6c), increased root volume (Figure 6d), a higher number of root branches (Figure 6e), as well as a greater projected root area (Figure 6f). These phenotypic traits further support the enhanced drought tolerance observed in the OE roots.

### GmNAC3 *Regulates Downstream Genes in Hairy Roots*

**Figure 7.**
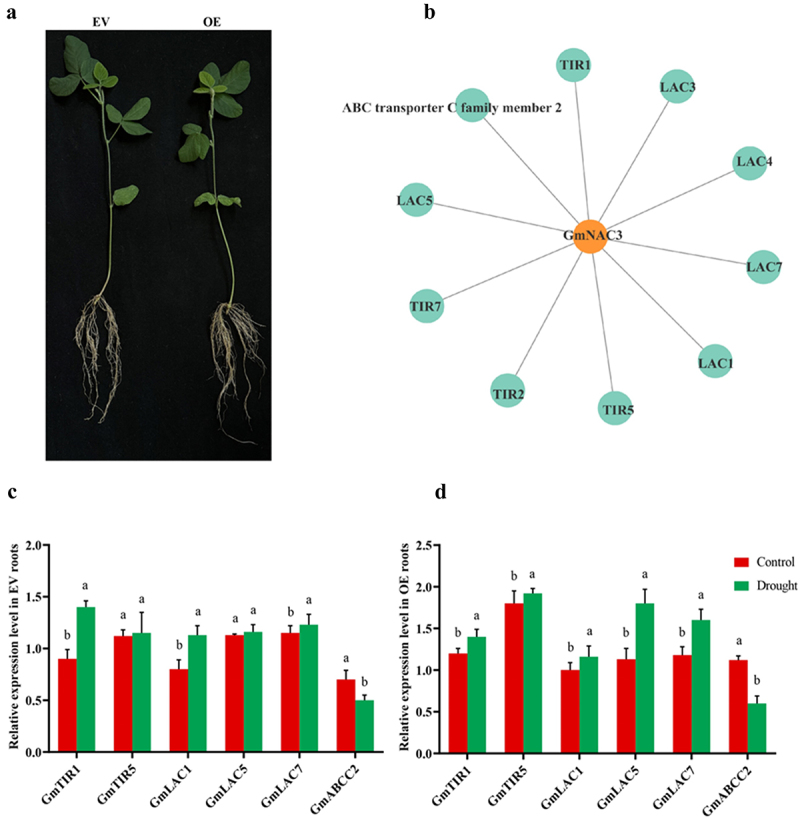
Expression analysis of the interacting genes of *GmNAC3*. (a) Image of EV and OE chimeric soybean plants. (b) PPI of *GmNAC3* TF. (c) Expression analysis in EV and OE roots. Different letters represent significant differences (p < .05).

To understand the regulatory network of *GmNAC3*, we identified ten predicted interacting genes using the STRING database (Figure 7b). The expression levels of six selected genes in response to PEG6000-induced drought stress were analyzed in EV and OE hairy roots via qRT-PCR. These genes were chosen for their potential involvement in auxin signaling, root development, and stress responses. *GmTIR1* and *GmTIR5*, which play key roles in auxin-regulated processes, showed slight upregulation in both EV and OE roots under drought stress (Figure 7c). Additionally, the *GmLAC* family, involved in lignin biosynthesis, displayed upregulation of *GmLAC5* and *GmLAC7* in OE roots under stress, while *GmLAC1* remained unchanged. Furthermore, *GmABCC2*, an ABC transporter gene, showed minor upregulation in EV roots but was significantly induced in OE roots under drought conditions (Figure 7d). These findings suggest that *GmNAC3* regulates a network of genes involved in stress adaptation.

### *Subcellular Localization of* GmNAC3

The subcellular localization of GmNAC3 was predicted using the mPLoc database, which indicated that it is located in the nucleus (Table S2). To confirm this prediction, a subcellular localization assay of the GmNAC3-GFP fusion protein was conducted in onion epidermal cells. The results demonstrated that GmNAC3 is indeed localized in the nucleus (Figure 7). Although a direct nuclear marker such as DAPI was not included in this assay, the punctate GFP signal observed at the central position of each cell, in contrast to the diffuse signal of the GFP control, strongly supports nuclear localization. This interpretation is consistent with the known characteristics of NAC transcription factors, which typically function in the nucleus. Nuclear-localized genes play critical roles in regulating signal transduction and gene expression, which are essential for plant growth, development, and stress responses.^35^ This finding suggests that *GmNAC3* might be involved in regulating various biological processes crucial for plant resilience to environmental stresses.

## Discussion

Soybean is a globally important crop, cultivated for various uses including food production, animal feed, pharmaceutical applications, and enhancing soil nitrogen levels.^36^ However, environmental stressors, especially drought, significantly hinder soybean growth and reduce crop yields. RNA-seq data have demonstrated that the overexpression (OE) of *GmWRKY54* and *GmWRKY12* in soybean enhances drought tolerance by improving stomatal regulation, ABA signaling, and the expression of various stress-related transcription factors.^37,38^ In *Arabidopsis*, *CAMTA1* knockout mutants exhibit increased drought sensitivity and altered expression of other drought-responsive transcription factors.^39^ *NAC* transcription factors are key regulators in plant responses to both biotic and abiotic stresses, as well as in various developmental processes.^6^ Although the NAC family has been extensively studied in various plants, the specific roles of *NAC* genes in soybean still require further exploration. Genome-wide studies have been conducted to investigate the *NAC* gene family in soybean^22,40–42^; however, the functional roles of many *NAC* members under stress conditions are yet to be fully elucidated. This study aims to investigate the role of the soybean *NAC3* gene under drought stress responses. Previous studies have highlighted *GmNAC3* as a key drought-responsive transcription factor in *Arabidopsis*.^5^ In chickpeas, *NAC3* genes exhibit organ-specific expression patterns, with induction linked to leaf aging and a significant upregulation in response to drought, ABA, ethephon (Et), and indole-3-acetic acid (IAA), while being downregulated by *N*-6-benzyl-adenine (6-BA).^43^ Building on these findings and our prior research in *Arabidopsis*, we selected *GmNAC3* for functional characterization in soybean hairy roots. We constructed an overexpression vector for *GmNAC3* and introduced it into soybean roots. Drought stress assays on chimeric soybean plants (OE-*GmNAC3*) revealed that these plants exhibited enhanced drought tolerance, outperforming non-transgenic controls. Under drought conditions, the survival rates of OE-*GmNAC3* plants were significantly higher than those of non-transgenic plants (Figure 2). Moreover, OE-*GmNAC3* plants displayed more lateral and longer roots compared to EV plants (Figure 3a). Root scanning of OE-*GmNAC3* plants also demonstrated superior growth metrics in response to drought stress relative to EV controls (Figure 3). This enhanced root development likely contributes to the improved drought resilience, as an efficient root system is critical for water uptake and plant adaptation under drought conditions.

**Figure 8.**
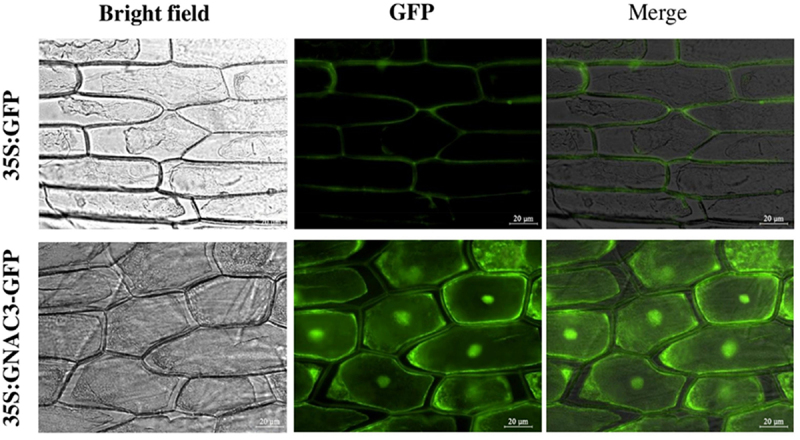
Subcellular localization of *GmNAC3* using transient expression assay in onion epidermis. Subcellular distribution of the 35S: GFP and 35S: *GmNAC3*-GFP in onion epidermal layer. Each row starts with a bright field image, followed by a GFP fluorescence image, and concludes with a merged fluorescence image. An automated fluorescence microscope was used to examine the fluorescence signals.

Drought stress elevates the production of reactive oxygen species (ROS), which can damage cellular structures and inhibit plant growth. To mitigate this oxidative stress, plants activate antioxidant systems to scavenge excess ROS.^44^ Drought conditions adversely affect photosynthesis, leading to increased levels of ROS, such as H₂O₂ and O₂^−^, which serve as key indicators of oxidative damage.^45^ In our study, OE-*GmNAC3* transgenic soybean plants exhibited reduced levels of H₂O₂ and O₂^−^ after drought stress (Figure 8), suggesting that *GmNAC3* overexpression helps regulate photosynthetic performance and limits ROS production. In rice, silencing the *OsPPS1* gene increased sensitivity to salt and ABA stress and led to elevated ROS accumulation.^46^ Similarly, overexpression of *MsMYB2L* in alfalfa enhanced the accumulation of osmoregulatory compounds such as proline and soluble sugars while reducing lipid peroxidation.^47^ In our experiments, OE-*GmNAC3* plants exhibited higher proline content and CAT activity, while REL and MDA levels were significantly reduced compared to control plants (Figure 4). These results indicate that *GmNAC3* plays a vital role in enhancing drought tolerance in soybean. The subcellular localization analysis of a gene is crucial for understanding its role in stress responses and protein synthesis.^48^ According to predictions from the plant mPLoc database, *GmNAC3* is expected to localize in the nucleus.^5^ To experimentally validate this prediction, we performed a transient expression assay in onion epidermal cells using a GFP fusion construct. The results confirmed that *GmNAC3* is indeed localized in the nucleus (Figure 8). Nuclear-localized genes are key to regulating signal transduction and gene expression, both of which are critical for plant growth, development, and stress responses.^49^ Thus, we proposed that *GmNAC3* might play a pivotal role in regulating biological processes essential for plant resilience to drought stress.

Auxin, a key phytohormone regulating root development, operates in coordination with NAC transcription factors, which also play vital roles in modulating this developmental process.^50,51^ In response to drought, ABA-responsive genes are regulated by transcription factors such as DREB, which interact with ABA-responsive element (ABRE) binding factors.^52^ Previous studies have shown that the *GmTIR1*, involved in auxin signaling, exhibits dynamic expression patterns during nodulation and stress responses. Overexpression of *GmTIR1* has been linked to enhanced germination and root elongation under drought and salt stress.^53^ Laccases (LACs) are multifunctional TFs that involved in lignin biosynthesis, plant development and stress responses.^54^ Moreover, ATP-binding cassette (ABC) transporters are critical for nutrient uptake, development, and abiotic stress responses.^55^ NAC genes also influence ABA biosynthesis and signaling. In *Arabidopsis*, *SNAC* and *ATAF1* interact with the *NCED3* promoter to regulate ABA production,^56^ while *AtNAC096* collaborates with *AREB* factors to respond to osmotic stress.^57^ Overexpression of *GmNAC3* in *Arabidopsis* upregulated stress-responsive transcription factors and ABA-related genes.^5^ To explore the mechanisms underlying the enhanced stress tolerance and root elongation observed in OE-*GmNAC3* plants, we analyzed the expression of six interacting genes, including *GmTIR1*, *GmTIR5*, *GmLAC1*, *GmLAC5*, *GmLAC7*, and *GmABC2*. The results revealed that *GmNAC3* overexpression led to the upregulation of *GmTIR5*, *GmLAC1*, *GmLAC5*, and *GmLAC7* compared to controls (Figure 6c,d). These findings are consistent with previous studies on *NAC31* overexpression in drought-stressed *Arabidopsis*, which also reported enhanced stress tolerance.^58^ These findings suggest that the overexpression of *GmNAC3* enhances drought tolerance by regulating key genes involved in stress signaling and root development. The overexpression of *GmNAC3* in soybean significantly improves drought tolerance at the phenotypic, physiological, and molecular levels. The soybean genome contains 179 NAC family, classified into 15 subgroups.^42^ *GmNAC3*, located on chromosome 1 and belonging to subgroup G, plays a crucial role in drought stress responses. We propose that *GmNAC3* functions as a key regulator that links ABA and auxin signaling pathways, coordinating the plant’s response to drought stress (Figure S3). This regulation likely influences root architecture and water acquisition, making *GmNAC3* a promising candidate for developing drought-resistant soybean varieties.

## Conclusion

The present study provides compelling evidence that *GmNAC3* is a critical regulator of drought tolerance in soybean. Overexpression of *GmNAC3* in chimeric soybean hairy roots resulted in noticeable improvements in physiological and molecular responses under PEG-induced drought stress, including enhanced biomass accumulation, reduced ROS levels, and upregulation of key stress-responsive genes such as *GmLAC5* and *GmLAC7*. These findings not only confirm the functional significance of *GmNAC3* in promoting drought resilience but also highlight its potential value as a promising genetic target for developing drought-tolerant soybean cultivars through biotechnological interventions.

## Supplementary Material

Supplementary file updated.docx

## Data Availability

The data utilized in this research is available within the article and its supplementary materials.
