# Supplementary material for: Transcriptional regulation of GmNAC3-mediated drought stress tolerance in soybean
Source: GM Crops Food. 2025 Jun 24;16(1):435–49. doi: 10.1080/21645698.2025.2516295 (PMC12758339; doi:10.1080/21645698.2025.2516295)
Supplement: Supplementary file updated.docx [file KGMC_A_2516295_SM1418.docx]

# **Transcriptional Regulation of *GmNAC3*-Mediated Drought Stress Tolerance in Soybean**

**Nooral Amin^1,2†^, Liu Lu ^1†^, Faizur Rehman^1^, Fei Chen^2^, Muhammad Imran^2^, Gai Yuhong*^1^, Piwu Wang^1,^* and Wei Jian^1,^***

1 Plant Biotechnology Centre, College of Agronomy, Jilin Agricultural University, Changchun 130118, China

2 School of Breeding and Multiplication (Sanya Institute of Breeding and Multiplication), School of Tropical Agriculture and Forestry, Hainan University, Sanya 572025, China

**>GmNAC3 (Glyma.01G051300)** **Gene CDS sequence: (840 bp)**

ATGAGTAAGATCAGCAACTTGAGTTCTGTAAGCAGTTCTGATCTCATAGATGCCAAGCTTGAAGAGCATCAGTTGTGTGGATCCAAGCAGTGCCCCGGTTGCGGACACAAGTTTGAAGGGAAGCCGGATTGGCTAGGCTTGCCCGCAGGAGTGAAGTTTGATCCCACAGACCAAGAACTAATAGAACATCTTGAAGCCAAAGTGGAGGCAAAGAACATGAAATCACACCCTTTGATAGATGAGTTCATTCCCACTATTGAAGGAGAAGATGGAATTTGTTATACCCATCCGGAGAAACTTCCAGGAGTAACAAGAGATGGGTTGAGCAGGCACTTCTTCCATAGACCTTCAAAGGCTTACACAACTGGGACAAGAAAGAGGAGAAAAATTCAAAACGAATGCGACTTGCAAGGTGGAGAAACCCGGTGGCACAAGACTGGTAAAACCAGGCCAGTGATGGTGAATGGAAAACAAAAGGGTTGCAAGAAAATTCTTGTCCTCTACACCAATTTCGGAAAGAACAGAAAACCTGAGAAAACCAACTGGGTGATGCACCAATACCATTTGGGGCAGCATGAGGAAGAGAAAGAAGGAGAGCTTGTGGTGTCAAAGATATTCTATCAGACACAGCCAAGGCAGTGCAATTGGTCAGATAGAAGTGCAACAACTGGTGAAGGAAGTGGAGAACCTAACAACAGTGGTAGAAGGGACAGTGGAAGTGGAAGTTGTTCTTCTAAGGAAATTGTTACTCACAGAGATGAGATGTCTGCTGTTGTTGGTGTCCCTCCAATGACAAGTTTCACTCATCATCATCCCTTGGATATTCAACAGCTAAAACCTGATCACTTCAGCTTCATCCCTTTCAGGAAAAGCTTTGATGAGGTTGGAATAGGAGAGGCTTCAACAGCAAGAGAAGTAATGCAAGCATCAGGTTCATGTGAAGAAGTGCATGAACGGCACCTAGCACAAGTAACTCCTCATCATCATCAACTACAACAACATGCTCATCATCATCAAATTTCAAACTCAGCTTTTCATATTAGTAGGCCGTCACATCCCATCTCTACCATTATCTCTCCTCCTCCCCTCCACCACACTTCCATCATTCTCGATGACAACTCTTACCATGTCTCTAGAATAATGCTCCAAAATGAAAATTTTCAGCAACAGCAGCAACAACATCATAAGCTTGGAGGAAGGTCTGCGTCTGGTTTGGAGGAACTCATAATGGGTTGCACTTCAACTGAAATCAAAGAGGAGTCATCCATCACAAATGCACAAGAAGCTGAATGGTTGAAGTACTCTTCTTATTGGCCAGACCCTGACAACCAGGATCATCATGGGTAG

**>GmNAC3 Protein sequence (326 bp)**

MENRTSSVLPPGFRFHPTDEELIVYYLCNQASSRPCPASIIPEVDIYKFDPWELPDKTDFGEKEWYFFSPRERKYPNGVRPNRATVSGYWKATGTDKAIYSGSKHVGVKKALVFYKGKPPKGLKTDWIMHEYRLIGSRRQANRQVGSMRLDDWVLCRIYKKKNIGKSMEAKEDYPIAQINLTPANNNSEQELVKFPRTSSLTHLLEMDYLGPISHILPDASYNSTFDFQINTANGGIDPFVKPQLVEIPYATDSGKYQVKQNSTINPTIFVNQVYDQRG

Table S1. Primers for the qRT-PCR of *GmNAC3* regulatory network in soybean.

| No. | Gene name | Primer | Primer Sequence (5’to 3‘) | Bp |
| --- | --- | --- | --- | --- |
| 1 | *GmTIR-1* | F  R | TGGGTTTAGGGAGTTTTGGG  TGTAGCTTATGACAGTGCCG | 20  20 |
| 2 | *GmTIR-5* | F  R | AATCCCATGTAACCAACGAGC  TAGCCTTCCTCCATAAACCTACC | 21  23 |
| 3 | *GmLaccease-1* | F  R | CCGAAAAGGTACAACTTGGTG  TGCTAAACCCCACGAAGTATG | 20  20 |
| 4 | *GmLaccease-5* | F  R | ACCATCCCATTCATCTACACG  CCATTAACAGGTACACCGACAG | 21  22 |
| 5 | *GmLaccease-7* | F  R | GATTCTACTCCAACCTCACTGC  GAAGCTGAGAATTTTGGACACG | 22  22 |
| 6 | *GmABCC-2* | F  R | GAGCAACCAAATCCCATGTAC  CTACCCATAAAGCCTTCCTCAC | 21  22 |
| 7 | *AtActin11* | F  R | ACCGGAATGGTTAAGGCTGG  ACTGAGCCTCATCACCAACG | 20  20 |
| 8 | *BAR* | F  R | TCAAATCTCGGTGACGGGC  GTCTGCACCATCGTCAACCACTA | 20  23 |


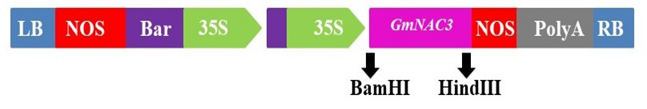

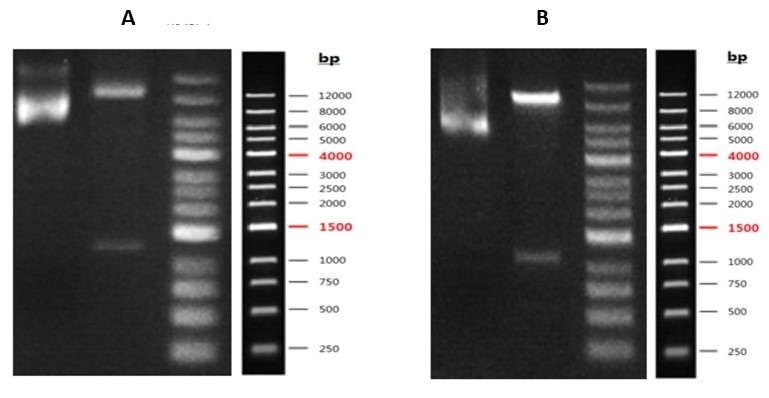

**Figure S1.** Construction of plant overexpression vector. Schematic illustration of the expression vector. (A) Digestion with BamHI. Lane 1: plasmid, Lane 2: plasmid digested with BamH1 (8820/1229), and Lane 3: (B) DNA Marker. Digestion with Hind111. Lane 1: plasmid, Lane 2: plasmid digested with Hind111(9198/1076), Lane 3: DNA marker.


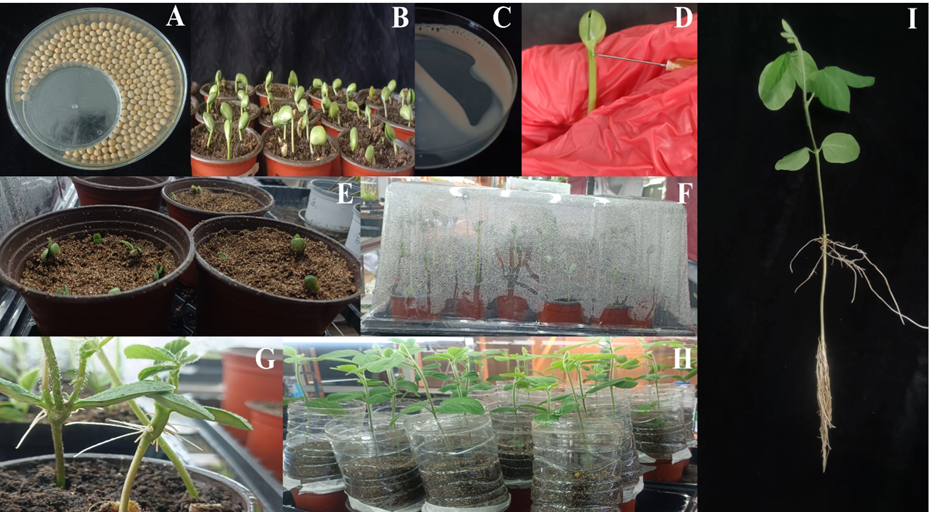


**Figure S2.** Different stages of the soybean hairy root transformation. (A) Seed sterilization process. (B) The optimal stage for transformation. (C) Inoculation of bacterial paste onto the plant. (D) Injection of bacterial paste into the cotyledonary node and upper hypocotyl. (E) Infected seedlings were transplanted into humidified vermiculite. (F) Infected seedlings transferred to a vent-controlled lid tray for further growth. (G) Appearance of developing hairy roots. (H) After 12 days of inoculation, seedlings were covered with additional humidified vermiculite to promote root regeneration. (I) Transformed soybean plant exhibiting chimeric roots.


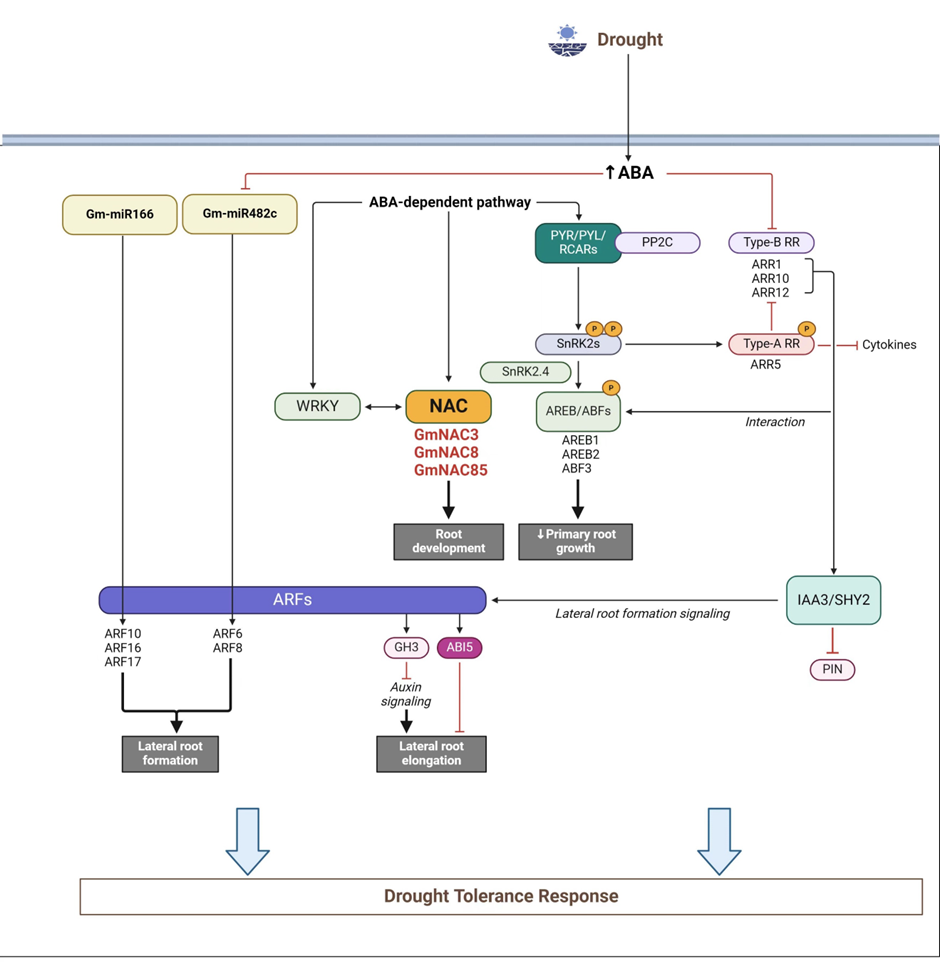


**Figure S3**. The regulatory model demonstrates the role of *NAC3* transcription factor via ABA dependent pathway. It showed that *NAC3* could interact with other key regulators, such as miRNAs and auxin signaling pathways, in developing a drought tolerant response in soybean.

**Figure S4**. Diagram of the molecular components of the cloning vector.
(pCAMBIAI3301 plant overexpression vector).


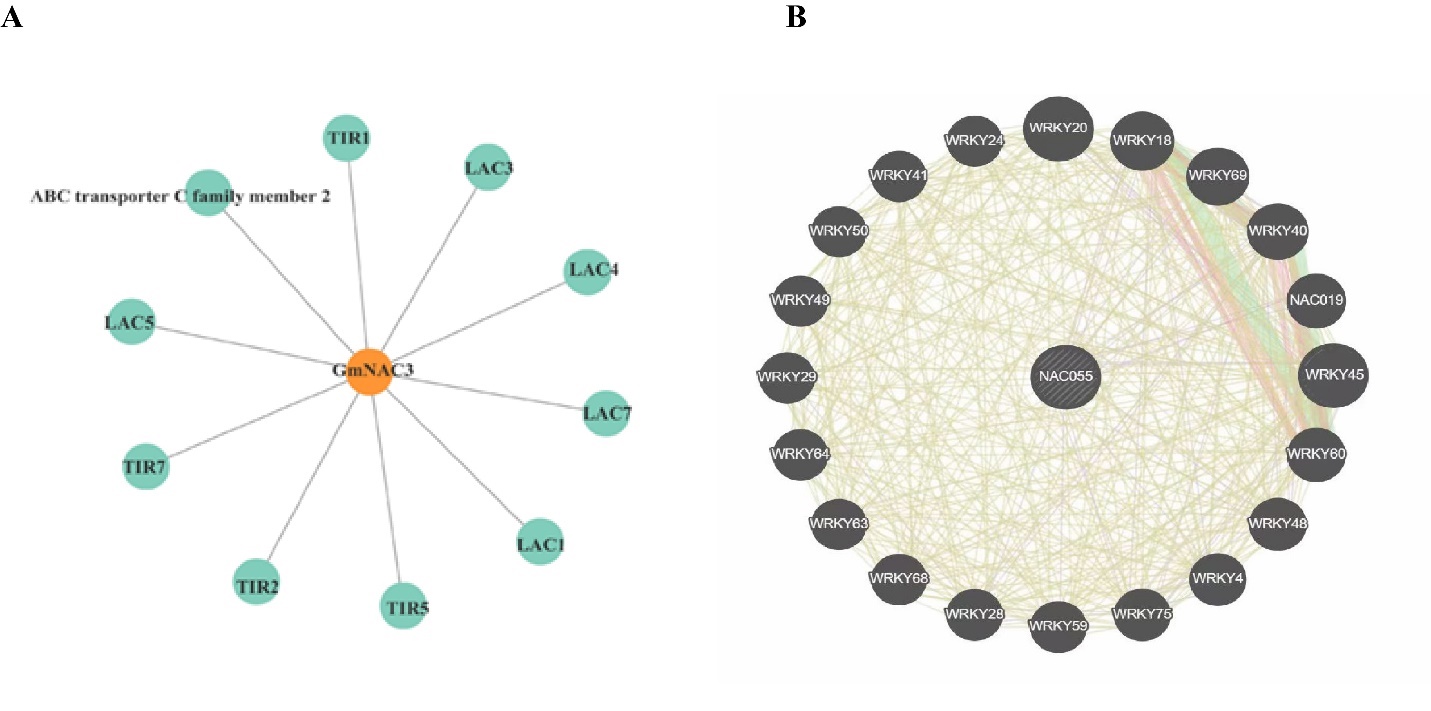
**Figure S5**. Protein protein interaction (PPI) network of *GmNAC3* in soybean using STRING database. (B) Interactive prediction of the of *GmNAC3* regulatory network in Arabidopsis orthologue *(AtNAC055)*  through the GeneMANIA database.

**Supplementary Table S2.** Physicochemical properties of *GmNAC* encoding proteins.

| **Name** | **Locus Id** | **Chr** | **Start** | **End** | **AA** | **Mw (kDa)** | **PI** | **SL** |
| --- | --- | --- | --- | --- | --- | --- | --- | --- |
| *GmNAC1* | Glyma.01G005500 | Gm01 | 544871 | 550997 | 451 | 50981.88 | 6.64 | N |
| *GmNAC2* | Glyma.01G046800 | Gm01 | 5448322 | 5450706 | 438 | 49723.09 | 5.95 | N |
| *GmNAC3* | Glyma.01G051300 | Gm01 | 6049546 | 6051316 | 279 | 31988.31 | 9.01 | N |
| *GmNAC4* | Glyma.01G088200 | Gm01 | 26763834 | 26769049 | 451 | 50919.95 | 4.94 | N |
| *GmNAC5* | Glyma.01G167900 | Gm01 | 51644827 | 51649975 | 344 | 39585.29 | 5.14 | N |
| *GmNAC6* | Glyma.02G050100 | Gm02 | 4519261 | 4523376 | 362 | 42022.27 | 6.10 | N |
| *GmNAC7* | Glyma.02G070000 | Gm02 | 6128068 | 6130838 | 354 | 40900.27 | 8.51 | N |
| *GmNAC8* | Glyma.02G070600 | Gm02 | 6188441 | 6190755 | 410 | 46762.43 | 6.33 | N |
| *GmNAC9* | Glyma.02G100200 | Gm02 | 9440858 | 9446878 | 436 | 48534.05 | 5.14 | N |
| *GmNAC10* | Glyma.02G107000 | Gm02 | 10198744 | 10201116 | 442 | 50123.54 | 6.21 | N |
| *GmNAC11* | Glyma.02G109800 | Gm02 | 10525394 | 10527411 | 279 | 32055.41 | 9.00 | N |
| *GmNAC12* | Glyma.02G222300 | Gm02 | 40991314 | 40995436 | 589 | 65601.58 | 4.88 | N |
| *GmNAC13* | Glyma.02G240500 | Gm02 | 42900252 | 42904927 | 643 | 73271.22 | 5.26 | N |
| *GmNAC14* | Glyma.02G284300 | Gm02 | 46586195 | 46589611 | 320 | 36904.54 | 8.57 | N |
| *GmNAC15* | Glyma.03G164200 | Gm03 | 37902284 | 37909534 | 296 | 34001.01 | 5.77 | N |
| *GmNAC16* | Glyma.03G179600 | Gm03 | 39180450 | 39181924 | 287 | 33515.15 | 8.61 | N |
| *GmNAC17* | Glyma.03G197900 | Gm03 | 40721164 | 40723468 | 257 | 29481.98 | 6.40 | N |
| *GmNAC18* | Glyma.04G014900 | Gm04 | 1134897 | 1137831 | 350 | 39922.28 | 7.72 | N |
| *GmNAC19* | Glyma.04G078600 | Gm04 | 6592149 | 6595131 | 357 | 39811.90 | 8.83 | N |
| *GmNAC20* | Glyma.04G119500 | Gm04 | 14396059 | 14401920 | 341 | 39855.46 | 5.82 | N |
| *GmNAC21* | Glyma.04G167200 | Gm04 | 41938604 | 41940695 | 357 | 40474.50 | 8.68 | N |
| *GmNAC22* | Glyma.04G175800 | Gm04 | 43891286 | 43891596 | 78 | 9020.34 | 9.13 | N |
| *GmNAC23* | Glyma.04G199000 | Gm04 | 47121567 | 47121567 | 169 | 18797.79 | 5.38 | N |
| *GmNAC24* | Glyma.04G208300 | Gm04 | 48062886 | 48064803 | 291 | 33654.12 | 6.41 | N |
| *GmNAC25* | Glyma.04G212000 | Gm04 | 48385896 | 48387157 | 201 | 23157.98 | 4.94 | N |
| *GmNAC26* | Glyma.04G213300 | Gm04 | 48507581 | 48512003 | 483 | 52632.71 | 4.81 | N |
| *GmNAC27* | Glyma.04G226700 | Gm04 | 49623094 | 49626512 | 603 | 67628.58 | 5.25 | N |
| *GmNAC28* | Glyma.04G249000 | Gm04 | 51588779 | 51591690 | 300 | 34310.10 | 8.60 | N |
| *GmNAC29* | Glyma.05G002700 | Gm05 | 192322 | 197211 | 390 | 43972.78 | 5.14 | N |
| *GmNAC30* | Glyma.05G025500 | Gm05 | 2217936 | 2219956 | 350 | 39483.53 | 7.08 | N |
| *GmNAC31* | Glyma.05G055900 | Gm05 | 5069618 | 5076268 | 364 | 42302.87 | 6.41 | N |
| *GmNAC32* | Glyma.05G086000 | Gm05 | 15993921 | 15994598 | 177 | 20657.56 | 9.39 | N |
| *GmNAC33* | Glyma.05G113000 | Gm05 | 29996339 | 29999120 | 345 | 38904.40 | 5.66 | N |
| *GmNAC34* | Glyma.05G120500 | Gm05 | 31355106 | 31357014 | 189 | 21956.39 | 4.77 | N |
| *GmNAC35* | Glyma.05G191300 | Gm05 | 37700092 | 37702428 | 317 | 35026.56 | 4.78 | N |
| *GmNAC36* | Glyma.05G192500 | Gm05 | 37775249 | 37776479 | 206 | 23046.91 | 4.73 | N |
| *GmNAC37* | Glyma.05G195000 | Gm05 | 37939652 | 37941553 | 298 | 34223.73 | 6.46 | N |
| *GmNAC38* | Glyma.05G202300 | Gm05 | 38582712 | 38584640 | 241 | 27227.64 | 9.20 | N |
| *GmNAC39* | Glyma.05G225100 | Gm05 | 40387813 | 40393404 | 448 | 50540.33 | 6.83 | N |
| *GmNAC40* | Glyma.05G234200 | Gm05 | 41133563 | 41137142 | 321 | 36343.71 | 7.77 | N |
| *GmNAC41* | Glyma.06G014900 | Gm06 | 1116201 | 1118815 | 374 | 42776.00 | 6.81 | N |
| *GmNAC42* | Glyma.06G080200 | Gm06 | 6156955 | 6159890 | 355 | 39576.67 | 8.48 | N |
| *GmNAC43* | Glyma.06G114000 | Gm06 | 9242093 | 9244402 | 299 | 34049.61 | 6.25 | N |
| *GmNAC44* | Glyma.06G138100 | Gm06 | 1272268 | 11275624 | 598 | 67097.03 | 5.72 | N |
| *GmNAC45* | Glyma.06G152900 | Gm06 | 12478823 | 12482337 | 503 | 54466.89 | 4.82 | N |
| *GmNAC46* | Glyma.06G154400 | Gm06 | 12584515 | 12585942 | 204 | 23658.40 | 4.65 | N |
| *GmNAC47* | Glyma.06G157400 | Gm06 | 12937296 | 12939138 | 295 | 34173.54 | 6.10 | N |
| *GmNAC48* | Glyma.06G166500 | Gm06 | 13869494 | 13871353 | 248 | 28074.23 | 8.74 | N |
| *GmNAC49* | Glyma.06G195500 | Gm06 | 17486456 | 17488546 | 357 | 40199.38 | 8.82 | N |
| *GmNAC50* | Glyma.06G236000 | Gm06 | 38358744 | 38361292 | 375 | 41599.42 | 6.73 | N |
| *GmNAC51* | Glyma.06G248900 | Gm06 | 42013055 | 42015422 | 337 | 37897.83 | 6.46 | N |
| *GmNAC52* | Glyma.06G249100 | Gm06 | 42079579 | 42082464 | 368 | 40939.24 | 6.26 | N |
| *GmNAC53* | Glyma.06G288500 | Gm06 | 47712486 | 47716034 | 285 | 32385.43 | 8.41 | N |
| *GmNAC54* | Glyma.06G318900 | Gm06 | 50757688 | 50763325 | 363 | 42457.42 | 6.03 | N |
| *GmNAC55* | Glyma.07G047900 | Gm07 | 4016419 | 4019905 | 497 | 56740.62 | 5.77 | N |
| *GmNAC56* | Glyma.07G048000 | Gm07 | 4026951 | 4029750 | 405 | 45872.12 | 4.88 | N |
| *GmNAC57* | Glyma.07G048100 | Gm07 | 4042093 | 4044551 | 308 | 34914.34 | 5.37 | N |
| *GmNAC58* | Glyma.07G050600 | Gm07 | 4330571 | 4333242 | 400 | 45045.50 | 6.11 | N |
| *GmNAC59* | Glyma.07G092000 | Gm07 | 8597242 | 8600354 | 324 | 36387.26 | 8.15 | N |
| *GmNAC60* | Glyma.07G126500 | Gm07 | 15101055 | 15107128 | 447 | 50324.13 | 6.60 | N |
| *GmNAC61* | Glyma.07G192900 | Gm07 | 36076875 | 36079545 | 362 | 40535.11 | 7.24 | N |
| *GmNAC62* | Glyma.07G201800 | Gm07 | 37074546 | 37078552 | 326 | 37090.56 | 6.43 | N |
| *GmNAC63* | Glyma.07G229100 | Gm07 | 40913721 | 40915908 | 233 | 26671.39 | 8.21 | N |
| *GmNAC64* | Glyma.07G271100 | Gm07 | 44367278 | 44369691 | 400 | 45500.06 | 6.71 | N |
| *GmNAC65* | Glyma.08G009700 | Gm08 | 753940 | 756150 | 241 | 27331.75 | 9.20 | N |
| *GmNAC66* | Glyma.08G031900 | Gm08 | 2547941 | 2553643 | 452 | 50960.81 | 6.80 | N |
| *GmNAC67* | Glyma.08G041500 | Gm08 | 3290821 | 3294321 | 313 | 35222.47 | 8.84 | N |
| *GmNAC68* | Glyma.08G075300 | Gm08 | 5759118 | 5761050 | 190 | 22112.62 | 4.85 | N |
| *GmNAC69* | Glyma.08G156500 | Gm08 | 12100644 | 12103960 | 323 | 35802.16 | 4.81 | N |
| *GmNAC70* | Glyma.08G161300 | Gm08 | 12508840 | 12512499 | 328 | 37775.40 | 6.83 | N |
| *GmNAC71* | Glyma.08G163100 | Gm08 | 12786187 | 12790560 | 348 | 39940.94 | 4.89 | N |
| *GmNAC72* | Glyma.08G169400 | Gm08 | 13802696 | 13807486 | 80 | 9604.85 | 4.81 | N |
| *GmNAC73* | Glyma.08G173400 | Gm08 | 13802696 | 13807486 | 302 | 34357.10 | 8.04 | N |
| *GmNAC74* | Glyma.08G181100 | Gm08 | 14538880 | 14540424 | 190 | 21917.45 | 5.14 | N |
| *GmNAC75* | Glyma.08G301100 | Gm08 | 41913160 | 41916084 | 413 | 46713.96 | 8.57 | N |
| *GmNAC76* | Glyma.08G307100 | Gm08 | 42523558 | 42529971 | 304 | 35439.34 | 8.51 | N |
| *GmNAC77* | Glyma.08G360200 | Gm08 | 47210855 | 47212576 | 224 | 25619.01 | 9.36 | N |
| *GmNAC78* | Glyma.09G167400 | Gm09 | 39166685 | 39170373 | 265 | 30871.74 | 5.64 | N |
| *GmNAC79* | Glyma.09G184100 | Gm09 | 40919062 | 40921935 | 331 | 37151.13 | 8.56 | N |
| *GmNAC80* | Glyma.09G231700 | Gm09 | 45495672 | 45500678 | 354 | 41019.15 | 6.14 | N |
| *GmNAC81* | Glyma.09G233600 | Gm09 | 45626675 | 45630038 | 359 | 41277.00 | 7.70 | N |
| *GmNAC82* | Glyma.09G235700 | Gm09 | 45833117 | 45837361 | 363 | 41119.16 | 5.75 | N |
| *GmNAC83* | Glyma.10G037700 | Gm10 | 3341593 | 3347333 | 296 | 33840.89 | 6.41 | N |
| *GmNAC84* | Glyma.10G077000 | Gm10 | 8257526 | 8258068 | 128 | 14785.89 | 9.43 | N |
| *GmNAC85* | Glyma.10G077400 | Gm10 | 8403343 | 8403900 | 136 | 15780.02 | 9.46 | N |
| *GmNAC86* | Glyma.10G197500 | Gm10 | 42882331 | 42888608 | 465 | 52044.40 | 6.85 | N |
| *GmNAC87* | Glyma.10G197600 | Gm10 | 42899234 | 42904478 | 448 | 49659.35 | 4.50 | N |
| *GmNAC88* | Glyma.10G204700 | Gm10 | 43595397 | 43599782 | 422 | 47399.00 | 5.29 | N |
| *GmNAC89* | Glyma.10G216400 | Gm10 | 44817031 | 44822075 | 346 | 39742.26 | 6.27 | N |
| *GmNAC90* | Glyma.10G219600 | Gm10 | 45126440 | 45130601 | 560 | 62759.89 | 4.70 | N |
| *GmNAC91* | Glyma.11G030600 | Gm11 | 2213842 | 2217833 | 360 | 41477.61 | 6.89 | N |
| *GmNAC92* | Glyma.11G075400 | Gm11 | 5649055 | 5653943 | 380 | 43860.42 | 5.66 | N |
| *GmNAC93* | Glyma.11G096600 | Gm11 | 7361789 | 7364386 | 302 | 34881.04 | 8.50 | N |
| *GmNAC94* | Glyma.11G182000 | Gm11 | 24908076 | 24910057 | 246 | 28409.74 | 6.11 | N |
| *GmNAC95* | Glyma.11G212400 | Gm11 | 30521509 | 30526668 | 672 | 75877.29 | 5.40 | N |
| *GmNAC96* | Glyma.12G003200 | Gm12 | 262438 | 265930 | 356 | 40969.69 | 7.69 | N |
| *GmNAC97* | Glyma.12G004900 | Gm12 | 376442 | 381099 | 347 | 40196.12 | 5.62 | N |
| *GmNAC98* | Glyma.12G022700 | Gm12 | 1660693 | 1663004 | 297 | 34334.52 | 8.51 | N |
| *GmNAC99* | Glyma.12G091200 | Gm12 | 7464025 | 7465647 | 248 | 28438.99 | 6.71 | N |
| *GmNAC100* | Glyma.12G118700 | Gm12 | 12366452 | 12369799 | 284 | 32321.35 | 8.75 | N |
| *GmNAC101* | Glyma.12G145100 | Gm12 | 19250825 | 19252602 | 180 | 20820.04 | 9.54 | N |
| *GmNAC102* | Glyma.12G148900 | Gm12 | 21124630 | 21127549 | 360 | 40050.49 | 8.27 | N |
| *GmNAC103* | Glyma.12G149100 | Gm12 | 21206948 | 21209333 | 340 | 37983.81 | 6.01 | N |
| *GmNAC104* | Glyma.12G160100 | Gm12 | 28382310 | 28382788 | 133 | 15387.84 | 9.65 | N |
| *GmNAC105* | Glyma.12G161700 | Gm12 | 30307162 | 30309990 | 366 | 40961.61 | 6.92 | N |
| *GmNAC106* | Glyma.12G171600 | Gm12 | 32745419 | 32747843 | 349 | 39983.66 | 6.96 | N |
| *GmNAC107* | Glyma.12G186200 | Gm12 | 34708678 | 34710549 | 244 | 27616.65 | 6.76 | N |
| *GmNAC108* | Glyma.12G186900 | Gm12 | 34753427 | 34763019 | 493 | 56117.87 | 9.14 | N |
| *GmNAC109* | Glyma.12G206900 | Gm12 | 36693869 | 36696890 | 279 | 31818.98 | 8.93 | N |
| *GmNAC110* | Glyma.12G221400 | Gm12 | 38060278 | 38062569 | 375 | 41277.21 | 8.85 | N |
| *GmNAC111* | Glyma.12G221500 | Gm12 | 38083562 | 38085675 | 345 | 38981.77 | 8.15 | N |
| *GmNAC112* | Glyma.12G226500 | Gm12 | 38629959 | 38632784 | 343 | 38411.97 | 7.73 | N |
| *GmNAC113* | Glyma.13G030900 | Gm13 | 10137790 | 10139654 | 268 | 30561.76 | 8.10 | N |
| *GmNAC114* | Glyma.13G062000 | Gm13 | 15953721 | 15955834 | 358 | 40711.46 | 6.02 | N |
| *GmNAC115* | Glyma.13G063300 | Gm13 | 16180287 | 16182579 | 380 | 43346.96 | 7.94 | N |
| *GmNAC116* | Glyma.13G174700 | Gm13 | 28912927 | 28916764 | 329 | 37350.81 | 6.40 | N |
| *GmNAC117* | Glyma.13G234700 | Gm13 | 34537748 | 34542139 | 332 | 37362.29 | 8.69 | N |
| *GmNAC118* | Glyma.13G243200 | Gm13 | 35262813 | 35264688 | 363 | 40637.11 | 8.41 | N |
| *GmNAC119* | Glyma.13G274300 | Gm13 | 37580356 | 37583227 | 352 | 39652.33 | 7.73 | N |
| *GmNAC120* | Glyma.13G279900 | Gm13 | 38124292 | 38125955 | 343 | 38666.43 | 8.11 | N |
| *GmNAC121* | Glyma.13G280000 | Gm13 | 38147198 | 38149541 | 375 | 41433.32 | 7.82 | N |
| *GmNAC122* | Glyma.13G294000 | Gm13 | 39352609 | 39355045 | 279 | 31849.04 | 8.82 | N |
| *GmNAC123* | Glyma.13G314600 | Gm13 | 40993081 | 40996703 | 371 | 41950.92 | 7.10 | N |
| *GmNAC124* | Glyma.13G315300 | Gm13 | 41041851 | 41044605 | 253 | 28450.64 | 8.85 | N |
| *GmNAC125* | Glyma.13G327600 | Gm13 | 42227446 | 42229767 | 349 | 39939.63 | 6.91 | N |
| *GmNAC126* | Glyma.14G030700 | Gm14 | 2226406 | 2230206 | 326 | 37654.50 | 8.72 | N |
| *GmNAC127* | Glyma.14G084300 | Gm14 | 7388630 | 7392398 | 278 | 31253.26 | 5.31 | N |
| *GmNAC128* | Glyma.14G140100 | Gm14 | 27453859 | 27457797 | 373 | 41593.10 | 8.54 | N |
| *GmNAC129* | Glyma.14G152700 | Gm14 | 33160828 | 33163204 | 280 | 31768.03 | 6.99 | N |
| *GmNAC130* | Glyma.14G189300 | Gm14 | 45407343 | 45411346 | 590 | 65849.93 | 4.88 | N |
| *GmNAC131* | Glyma.14G210000 | Gm14 | 47524327 | 47529700 | 644 | 73440.71 | 5.08 | N |
| *GmNAC132* | Glyma.15G051200 | Gm15 | 4025824 | 4027347 | 191 | 22066.58 | 5.14 | N |
| *GmNAC133* | Glyma.15G070300 | Gm15 | 5387760 | 5389683 | 354 | 39585.05 | 8.58 | N |
| *GmNAC134* | Glyma.15G078300 | Gm15 | 6020602 | 6024367 | 322 | 36190.74 | 6.54 | N |
| *GmNAC135* | Glyma.15G254000 | Gm15 | 48295488 | 48300322 | 303 | 34335.09 | 8.51 | N |
| *GmNAC136* | Glyma.15G257700 | Gm15 | 48735321 | 48738017 | 318 | 36938.97 | 6.89 | N |
| *GmNAC137* | Glyma.15G264100 | Gm15 | 49820324 | 49824883 | 347 | 39765.87 | 5.02 | N |
| *GmNAC138* | Glyma.15G266500 | Gm15 | 50221849 | 50225934 | 326 | 37509.27 | 6.62 | N |
| *GmNAC139* | Glyma.16G016400 | Gm16 | 1431637 | 1433520 | 267 | 30421.41 | 5.44 | N |
| *GmNAC140* | Glyma.16G016600 | Gm16 | 1450709 | 1455979 | 431 | 49684.82 | 4.91 | N |
| *GmNAC141* | Glyma.16G016700 | Gm16 | 1460102 | 1463552 | 400 | 45291.25 | 4.60 | N |
| *GmNAC142* | Glyma.16G019400 | Gm16 | 1725643 | 1728272 | 443 | 49410.54 | 6.35 | N |
| *GmNAC143* | Glyma.16G042900 | Gm16 | 4011699 | 4014098 | 407 | 46089.22 | 6.20 | N |
| *GmNAC144* | Glyma.16G043200 | Gm16 | 4037637 | 4039984 | 353 | 39812.09 | 8.87 | N |
| *GmNAC145* | Glyma.16G051800 | Gm16 | 4995968 | 4997910 | 216 | 24521.69 | 8.53 | N |
| *GmNAC146* | Glyma.16G069300 | Gm16 | 6914905 | 6920531 | 399 | 44665.47 | 4.85 | N |
| *GmNAC147* | Glyma.16G130200 | Gm16 | 28340797 | 28344577 | 362 | 41920.91 | 5.92 | N |
| *GmNAC148* | Glyma.16G151500 | Gm16 | 31198908 | 31201131 | 363 | 41824.45 | 8.51 | N |
| *GmNAC149* | Glyma.16G152100 | Gm16 | 31264479 | 31266877 | 410 | 46655.29 | 6.33 | N |
| *GmNAC150* | Glyma.16G217400 | Gm16 | 37450061 | 37453768 | 264 | 30698.59 | 6.06 | N |
| *GmNAC151* | Glyma.17G002800 | Gm17 | 318373 | 320984 | 403 | 45803.44 | 6.51 | N |
| *GmNAC152* | Glyma.17G101500 | Gm17 | 7978357 | 7980301 | 350 | 39490.65 | 7.62 | N |
| *GmNAC153* | Glyma.17G138100 | Gm17 | 11141925 | 11148576 | 366 | 42419.09 | 6.43 | N |
| *GmNAC154* | Glyma.17G154100 | Gm17 | 12962459 | 12965411 | 342 | 38461.74 | 5.30 | N |
| *GmNAC155* | Glyma.17G185000 | Gm17 | 23531754 | 23534400 | 217 | 24741.40 | 9.24 | N |
| *GmNAC156* | Glyma.17G240700 | Gm17 | 39617641 | 39620959 | 285 | 32141.47 | 7.94 | N |
| *GmNAC157* | Glyma.18G043900 | Gm18 | 3767587 | 3772179 | 678 | 76538.16 | 5.52 | N |
| *GmNAC158* | Glyma.18G110700 | Gm18 | 13087248 | 13092989 | 304 | 35308.27 | 8.74 | N |
| *GmNAC159* | Glyma.18G119300 | Gm18 | 14754240 | 14757164 | 401 | 45037.77 | 8.76 | N |
| *GmNAC160* | Glyma.18G261300 | Gm18 | 54718321 | 54723680 | 388 | 43930.47 | 5.80 | N |
| *GmNAC161* | Glyma.18G301500 | Gm18 | 57926526 | 57928866 | 229 | 26173.44 | 8.99 | N |
| *GmNAC162* | Glyma.19G002900 | Gm19 | 187517 | 192326 | 389 | 43677.69 | 5.73 | N |
| *GmNAC163* | Glyma.19G021900 | Gm19 | 2444946 | 2448347 | 367 | 41529.86 | 6.60 | N |
| *GmNAC164* | Glyma.19G024500 | Gm19 | 2893626 | 2895846 | 362 | 41382.19 | 6.23 | N |
| *GmNAC165* | Glyma.19G056400 | Gm19 | 9892895 | 9897664 | 398 | 44397.21 | 5.02 | N |
| *GmNAC166* | Glyma.19G097700 | Gm19 | 34293345 | 34295089 | 215 | 24286.37 | 5.26 | N |
| *GmNAC167* | Glyma.19G108800 | Gm19 | 36152702 | 36154505 | 336 | 37864.82 | 8.60 | N |
| *GmNAC168* | Glyma.19G109100 | Gm19 | 36236790 | 36239152 | 405 | 46038.09 | 6.37 | N |
| *GmNAC169* | Glyma.19G165600 | Gm19 | 42649393 | 42657126 | 294 | 33595.66 | 5.87 | N |
| *GmNAC170* | Glyma.19G180300 | Gm19 | 43905106 | 43907015 | 337 | 38609.60 | 6.13 | N |
| *GmNAC171* | Glyma.19G195800 | Gm19 | 45294380 | 45296644 | 254 | 29272.31 | 5.28 | N |
| *GmNAC172* | Glyma.19G259500 | Gm19 | 50313492 | 50317793 | 265 | 30624.77 | 6.60 | N |
| *GmNAC173* | Glyma.19G259700 | Gm19 | 50330586 | 50331745 | 265 | 30588.69 | 6.09 | N |
| *GmNAC174* | Glyma.20G033300 | Gm20 | 4521780 | 4523753 | 280 | 32132.63 | 8.15 | N |
| *GmNAC175* | Glyma.20G172100 | Gm20 | 40983077 | 40987397 | 549 | 61694.61 | 4.75 | N |
| *GmNAC176* | Glyma.20G175500 | Gm20 | 41284327 | 41289331 | 341 | 39194.76 | 6.31 | N |
| *GmNAC177* | Glyma.20G185800 | Gm20 | 42438478 | 42443399 | 442 | 49828.98 | 5.35 | N |
| *GmNAC178* | Glyma.20G192300 | Gm20 | 43096801 | 43100393 | 604 | 66939.28 | 4.82 | N |
| *GmNAC179* | Glyma.20G192500 | Gm20 | 43138131 | 43145708 | 465 | 52099.49 | 6.81 | N |

Physico-chemical properties of GmNAC family members in Glycine max. Coding Sequence: CDS, Amino Acid: AA, Molecular Weight: MW, Isoelectric Point: PI, Subcellular Location: SL, N: Nuclear.

**Supplementary Table S3.** Randomly selected *Cis-*acting elements in the promoter of *GmNAC* genes (2000 bp upstream of CDS) that are involved in several biological procesess and stress responses.

| Gene | Element | Sequence | Site | Length | Chain | Element function | Group |
| --- | --- | --- | --- | --- | --- | --- | --- |
| *GmNAC1* | CGTCA-motif | CGTCA | 370 | 5 | - | *cis-*acting regulatory element involved in the MeJA-responsiveness | Methyl jasmonate |
| *GmNAC1* | CGTCA-motif | CGTCA | 1482 | 5 | + | *cis-*acting regulatory element involved in the MeJA-responsiveness | Methyl jasmonate |
| *GmNAC1* | I-box | Ggataaggtg | 160 | 9 | + | part of a light responsive element | Light |
| *GmNAC1* | Box 4 | ATTAAT | 479 | 6 | + | part of a conserved DNA module involved in light responsiveness | Light |
| *GmNAC1* | Box 4 | ATTAAT | 485 | 6 | + | part of a conserved DNA module involved in light responsiveness | Light |
| *GmNAC1* | Box 4 | ATTAAT | 1570 | 6 | - | part of a conserved DNA module involved in light responsiveness | Light |
| *GmNAC1* | Box 4 | ATTAAT | 1724 | 6 | - | part of a conserved DNA module involved in light responsiveness | Light |
| *GmNAC1* | TGACG-motif | TGACG | 370 | 5 | + | *cis-*acting regulatory element involved in the MeJA-responsiveness | Methyl jasmonate |
| *GmNAC1* | TGACG-motif | TGACG | 1482 | 5 | - | *cis-*acting regulatory element involved in the MeJA-responsiveness | Methyl jasmonate |
| *GmNAC1* | Sp1 | GGGCGG | 281 | 6 | + | light responsive element | Light |
| *GmNAC1* | GATA-motif | GATAGGG | 161 | 7 | + | part of a light responsive element | Light |
| *GmNAC1* | O2-site | GATGATGTGG | 1479 | 9 | - | *cis-*acting regulatory element involved in zein metabolism regulation | Zein metabolism regulation |
| *GmNAC1* | G-box | CACGTC | 1480 | 6 | + | *cis-*acting regulatory element involved in light responsiveness | Light |
| *GmNAC1* | GT1-motif | GGTTAA | 1034 | 6 | + | light responsive element | Light |
| *GmNAC1* | ARE | AAACCA | 287 | 6 | - | *cis-*acting regulatory element essential for the anaerobic induction | Anaerobic |
| *GmNAC1* | ARE | AAACCA | 672 | 6 | - | *cis-*acting regulatory element essential for the anaerobic induction | Anaerobic |
| *GmNAC1* | ARE | AAACCA | 943 | 6 | - | *cis-*acting regulatory element essential for the anaerobic induction | Anaerobic |
| *GmNAC1* | MBS | CAACTG | 97 | 6 | + | MYB binding site involved in drought-inducibility | Drought |
| *GmNAC1* | TGA-element | AACGAC | 364 | 6 | + | auxin-responsive element | Auxin |
| *GmNAC1* | RY-element | CATGCATG | 1759 | 8 | - | *cis-*acting regulatory element involved in seed-specific regulation | Seed-specific regulation |
| *GmNAC1* | ATCT-motif | AATCTAATCC | 849 | 9 | - | part of a conserved DNA module involved in light responsiveness | Light |
| *GmNAC1* | ABRE | ACGTG | 1480 | 5 | - | *cis-*acting element involved in the abscisic acid responsiveness | Abscisic acid |
| *GmNAC3* | CGTCA-motif | CGTCA | 769 | 5 | - | *cis-acting regulatory element involved in the MeJA-responsiveness* | Methyl jasmonate |
| *GmNAC3* | CGTCA-motif | CGTCA | 1605 | 5 | + | *cis-acting regulatory element involved in the MeJA-responsiveness* | Methyl jasmonate |
| *GmNAC3* | TC-rich repeats | GTTTTCTTAC | 1133 | 9 | - | *cis-acting element involved in defense and stress responsiveness* | Defense and stress |
| *GmNAC3* | P-box | CCTTTTG | 484 | 7 | - | *gibberellin-responsive element* | Gibberellin |
| *GmNAC3* | MBS | CAACTG | 1191 | 6 | - | *MYB binding site involved in drought-inducibility* | Drought |
| *GmNAC3* | Box 4 | ATTAAT | 1496 | 6 | - | *part of a conserved DNA module involved in light responsiveness* | Light |
| *GmNAC3* | GA-motif | ATAGATAA | 1863 | 8 | + | *part of a light responsive element* | Light |
| *GmNAC3* | ABRE | GCCGCGTGGC | 507 | 9 | - | *cis-acting element involved in the abscisic acid responsiveness* | Abscisic acid |
| *GmNAC22* | AAAC-motif | CAATCAAAACCT | 237 | 11 | + | *light responsive element* | Light |
| *GmNAC22* | TCCC-motif | TCTCCCT | 726 | 7 | + | *part of a light responsive element* | Light |
| *GmNAC22* | TGACG-motif | TGACG | 1388 | 5 | - | *cis-acting regulatory element involved in the MeJA-responsiveness* | Methyl jasmonate |
| *GmNAC23* | CGTCA-motif | CGTCA | 65 | 5 | - | *cis-acting regulatory element involved in the MeJA-responsiveness* | Methyl jasmonate |
| *GmNAC23* | MBSI | aaaAaaC(G/C)GTTA | 1882 | 10.5 | - | *MYB binding site involved in flavonoid biosynthetic genes regulation* | Flavonoid biosynthetic genes regulation |
| *GmNAC23* | AE-box | AGAAACAA | 23 | 8 | - | *part of a module for light response* | Light |
| *GmNAC23* | GARE-motif | TCTGTTG | 1976 | 7 | + | *gibberellin-responsive element* | Gibberellin |
| *GmNAC39* | TCA-element | CCATCTTTTT | 1151 | 9 | - | *cis-acting element involved in salicylic acid responsiveness* | Salicylic acid |
| *GmNAC39* | TCA-element | CCATCTTTTT | 1236 | 9 | - | *cis-acting element involved in salicylic acid responsiveness* | Salicylic acid |
| *GmNAC39* | CGTCA-motif | CGTCA | 7 | 5 | - | *cis-acting regulatory element involved in the MeJA-responsiveness* | Methyl jasmonate |
| *GmNAC39* | CGTCA-motif | CGTCA | 10 | 5 | + | *cis-acting regulatory element involved in the MeJA-responsiveness* | Methyl jasmonate |
| *GmNAC76* | ACE | GACACGTATG | 1083 | 9 | - | *cis-acting element involved in light responsiveness* | Light |
| *GmNAC76* | CGTCA-motif | CGTCA | 521 | 5 | - | *cis-acting regulatory element involved in the MeJA-responsiveness* | Methyl jasmonate |
| *GmNAC76* | TC-rich repeats | GTTTTCTTAC | 1269 | 9 | - | *cis-acting element involved in defense and stress responsiveness* | Defense and stress |
| *GmNAC76* | TGACG-motif | TGACG | 521 | 5 | + | *cis-acting regulatory element involved in the MeJA-responsiveness* | Methyl jasmonate |
| *GmNAC81* | MBS | CAACTG | 666 | 6 | - | *MYB binding site involved in drought-inducibility* | Drought |
| *GmNAC81* | GA-motif | ATAGATAA | 994 | 8 | - | *part of a light responsive element* | Light |
| *GmNAC81* | ABRE | ACGTG | 312 | 5 | + | *cis-acting element involved in the abscisic acid responsiveness* | Abscisic acid |
| *GmNAC81* | ABRE | ACGTG | 642 | 5 | - | *cis-acting element involved in the abscisic acid responsiveness* | Abscisic acid |
| *GmNAC81* | ABRE | CGTACGTGCA | 1662 | 9 | + | *cis-acting element involved in the abscisic acid responsiveness* | Abscisic acid |
| *GmNAC81* | CGTCA-motif | CGTCA | 640 | 5 | + | *cis-acting regulatory element involved in the MeJA-responsiveness* | Methyl jasmonate |
| *GmNAC81* | CGTCA-motif | CGTCA | 1120 | 5 | - | *cis-acting regulatory element involved in the MeJA-responsiveness* | Methyl jasmonate |
| *GmNAC81* | CGTCA-motif | CGTCA | 1502 | 5 | + | *cis-acting regulatory element involved in the MeJA-responsiveness* | Methyl jasmonate |
